# Hemodynamic Assessment in Newborn Infants With Sepsis: A Prospective Observational Study

**DOI:** 10.7759/cureus.109826

**Published:** 2026-05-28

**Authors:** Tapan Singh, Shakal N Singh, Himanshu Gupta, Shalini Tripathi, Arpita Bhriguvanshi, Neeraj Anand, Manisha Verma, Akhil K Sharma

**Affiliations:** 1 Department of Paediatrics, King George's Medical University, Lucknow, IND; 2 Department of Cardiology, King George's Medical University, Lucknow, IND

**Keywords:** functional echocardiography, hemodynamic assessment, hemodynamic intervention, high mortality, low-birth-weight infant, neonatal sepsis, neonatal sequential organ failure assessment (nsofa), prematurity, septic shock, vasopressors

## Abstract

Background and objective: Sepsis is defined as SIRS (systemic inflammatory response syndrome) in the presence of confirmed or suspected infection. Neonatal sepsis remains a major cause of morbidity and mortality and is frequently complicated by septic shock, resulting in impaired tissue perfusion and organ dysfunction. Hemodynamic alterations in septic neonates are heterogeneous and often under-recognized. The primary objective of this study was to characterize the hemodynamic profile of neonates with sepsis for early detection of circulatory dysfunction using functional echocardiography. The secondary objective was to evaluate the requirement for vasoactive support and associated outcomes.

Methodology: This prospective observational study included 288 neonates with clinical sepsis admitted to the neonatal intensive care unit (NICU) at King George’s Medical University, Lucknow, India. Sepsis was diagnosed based on the presence of ≥2 clinical signs, suggestive of infection, including feeding intolerance, temperature instability, apnea, lethargy or poor reflexes, and capillary refill time greater than three seconds. These criteria were applied uniformly at the time of admission, and organ dysfunction was assessed using the neonatal Sequential Organ Failure Assessment (nSOFA) score. Septic shock was defined by clinical and hemodynamic evidence of circulatory failure requiring vasoactive support. Laboratory evaluation included sepsis screen, blood cultures, and cerebrospinal fluid analysis, where indicated. Hemodynamic assessment was performed using bedside functional two-dimensional echocardiography, evaluating preload, myocardial contractility, and afterload parameters.

Results: Of the 288 neonates with sepsis, 102 (35.4%) developed septic shock. Neonates with shock had significantly lower gestational age and birth weight, and a higher incidence of feeding intolerance and prolonged capillary refill time. Laboratory abnormalities included lower total leukocyte counts and higher C-reactive protein levels and immature-to-total neutrophil ratios (p < 0.001). Functional echocardiography demonstrated impaired myocardial contractility and altered perfusion parameters in the shock group. Overall, 80.4% of neonates with septic shock required inotropes and/or vasopressors. Mortality was significantly higher among neonates with shock compared to those without shock (46.1% vs. 9.1%). Blood and cerebrospinal fluid cultures predominantly yielded gram-negative organisms, with *Klebsiella pneumoniae* and *Acinetobacter baumannii* being the most common isolates.

Conclusion: Septic shock is common among neonates with sepsis, particularly in preterm and low-birth-weight infants, and is associated with significant hemodynamic compromise and high mortality. Early recognition of circulatory dysfunction using a combination of clinical assessment, laboratory markers, and functional echocardiography allows timely, targeted hemodynamic interventions with fluids, inotropes, and vasopressors, potentially improving neonatal outcomes.

## Introduction

Sepsis remains a leading cause of morbidity, mortality, and healthcare utilization among children worldwide, with the burden being disproportionately high in neonates. Globally, an estimated 2,202 cases of neonatal sepsis occur per 100,000 live births, contributing substantially to the nearly 1.2 million cases of childhood sepsis reported annually [1]. Neonates account for a significant proportion of sepsis-related hospitalizations and deaths, particularly in low- and middle-income countries (LMICs). Mortality associated with neonatal sepsis varies widely, ranging from 10% to over 50%, depending on gestational age, birth weight, severity of illness, and resource availability [2-9]. Most sepsis-related deaths in neonates occur due to refractory shock and/or multiple organ dysfunction syndrome (MODS), often within the first 48-72 hours of illness onset, underscoring the importance of early recognition and targeted management [10-13].

Neonatal sepsis presents a diagnostic challenge due to its non-specific clinical manifestations, which often overlap with non-infectious conditions [14,15]. Blood culture, although considered the gold standard for identifying bloodstream infections, has limited sensitivity, and even expert retrospective adjudication of suspected infection episodes shows poor interobserver agreement [15,16]. To standardize diagnosis, the International Paediatric Sepsis Consensus Conference (2002) introduced age-specific definitions for systemic inflammatory response syndrome (SIRS), sepsis, and septic shock in children, including criteria applicable to term neonates, consistent with the definitions provided by Goldstein et al. [16,17]. According to these definitions, SIRS is diagnosed when at least two of four clinical criteria are present (abnormal core temperature, WBC count deviations, abnormal heart rate, or respiratory rate), one of which must be abnormal temperature or leukocyte count [16]. Sepsis was defined as SIRS occurring in the presence of suspected or proven infection. Although widely used, SIRS-based definitions have important limitations in neonates because inflammatory responses may be subtle, blunted, or atypical, leading to increasing emphasis on organ dysfunction-based assessment systems such as neonatal Sequential Organ Failure Assessment (nSOFA) [18-20]. Recent studies suggest that organ dysfunction-based criteria may better identify severe sepsis and predict outcomes in critically ill neonates and children.

Septic shock, defined as sepsis-induced cardiovascular dysfunction due to hypovolemia, vasoregulatory failure, or myocardial dysfunction, represents the most severe end of the disease spectrum [17]. At a cellular level, shock reflects an imbalance between oxygen delivery and demand resulting from a complex interaction of hypovolemia, vasoregulatory failure, and myocardial dysfunction. In neonates, these processes are further influenced by unique physiological characteristics, including immature myocardial contractility, limited ability to augment stroke volume, transitional circulation, and reduced cardiovascular reserve. As a result, the hemodynamic manifestations of neonatal septic shock are highly variable and incompletely understood.

Most current knowledge regarding septic shock hemodynamics is derived from adult and older paediatric populations. Adult septic shock is typically characterized by reduced systemic vascular resistance with preserved or increased cardiac output, whereas paediatric septic shock demonstrates heterogeneous patterns, including both hyperdynamic and hypodynamic states [17,21-23]. Neonatal data are particularly sparse and are largely extrapolated from animal studies or small human cohorts, primarily involving late-onset sepsis [24]. Consequently, management strategies for neonatal septic shock remain largely empirical and extrapolated from older age groups, despite fundamental physiological differences.

Outcomes in paediatric and neonatal sepsis have improved over time, particularly following the introduction of protocolized care. The American College of Critical Care Medicine (ACCM) published Clinical Practice Parameters for Hemodynamic Support of Paediatric and Neonatal Shock in 2002, with subsequent updates in 2007, reporting markedly reduced mortality in selected populations receiving early, goal-directed therapy [17]. However, these outcomes reflect controlled settings and previously healthy cohorts, and mortality in real-world neonatal septic shock remains substantial, highlighting the need for improved bedside assessment and individualized management.

Traditionally, invasive arterial and pulmonary vascular catheterization has been used to evaluate hemodynamic status. In neonates, however, technical complexity, time constraints, and clinical instability often limit the feasibility of these methods. Advances in bedside functional echocardiography now provide a reliable, non-invasive, point-of-care alternative for real-time assessment of preload, myocardial contractility, and afterload in critically ill neonates. This approach enables early identification of circulatory dysfunction and supports individualized, physiology-guided therapeutic interventions.

Comprehensive hemodynamic assessment is, thus, central to the management of neonatal sepsis. Early identification of perfusion abnormalities using a combination of clinical evaluation, laboratory markers, and functional echocardiography may facilitate timely optimization of fluid therapy, inotropic support, and vasopressor use. The present study aims to assess hemodynamic alterations in neonates with sepsis to enable early detection of circulatory compromise and guide targeted management strategies, thereby improving clinical outcomes.

## Materials and methods

This was a prospective observational study conducted from November 1, 2022, to October 31, 2023, at the Neonatal Unit at Queen Mary’s Hospital and the Neonatal Intensive Care Unit (NICU) at King George’s Medical University (KGMU), Lucknow, India. Ethical approval was obtained from the King George's Medical University Institutional Ethics Committee (reference code XIV-PGTSC-IIA/P54). Written informed consent was obtained from parents or legal guardians prior to enrollment. The study was conducted in accordance with the principles of the Declaration of Helsinki, and confidentiality of patient data was maintained throughout.

Study population

All enrolled neonates fulfilled the criteria for clinical sepsis at admission. Neonates were eligible if they were admitted to the NICU with clinical features suggestive of sepsis, with or without cardiovascular compromise or requirement for respiratory support. The study also included premature neonates who were empirically started on antibiotics for suspected sepsis based on clinical presentation and/or risk factors while evaluation for sepsis was underway. Neonates whose parents declined consent, those with severe perinatal asphyxia (HIE stage III), or major congenital malformations were excluded.

A total of 288 neonates were enrolled. The minimum sample size was calculated using the formula $N = \frac{4Z^2P(1 - P)}{W^2}$, assuming a prevalence of septic shock among neonatal sepsis cases of 25% [25], an absolute precision of 5%, and a 95% confidence interval, resulting in a required sample size of 288. All consecutive neonates fulfilling the inclusion criteria during the study period were enrolled until the required sample size was achieved. The calculated minimum sample size was achieved without adjustment for attrition, as all enrolled neonates completed the study protocol. 

Definitions

Neonatal sepsis was defined by the presence of two or more clinical signs suggestive of infection, including feeding intolerance, temperature instability, apnea, lethargy or poor reflexes, and capillary refill time greater than three seconds [26]. These criteria were applied uniformly at the time of admission.

Septic shock was defined as sepsis-associated cardiovascular dysfunction characterized by persistent hypotension or signs of impaired perfusion (prolonged capillary refill, oliguria, metabolic acidosis) requiring treatment with inotropes and/or vasopressors, with or without fluid resuscitation, in accordance with International Paediatric Sepsis Consensus guidelines.

Assessments

Organ Dysfunction Assessment

The neonatal Sequential Organ Failure Assessment (nSOFA) score is used to evaluate organ dysfunction and predict ICU admission or mortality risk [18,27]. The score includes respiratory, cardiovascular, and hematological components and was assessed after admission to the NICU during the first 72 hours of life. Organ dysfunction assessment was performed for all enrolled neonates using the nSOFA score.

Respiratory scores ranged from 0 to 8, with 0 = not intubated or SpO₂/FiO₂ >300; 2 = intubated, SpO₂/FiO₂ <300; 4 = intubated, SpO₂/FiO₂ <200; 6 = intubated, SpO₂/FiO₂ <150; 8 = intubated, SpO₂/FiO₂ <100. Cardiovascular scores ranged from 0 to 4, with 0 = no inotropes or corticosteroids; 1 = no inotropes, corticosteroid treatment; 2 = 1 inotrope, no corticosteroids; 3 = >2 inotropes or 1 inotrope with corticosteroids; 4 = >2 inotropes with corticosteroids. Hematologic scores ranged from 0 to 3, with 0 = platelets >150 × 10³/µL; 1 = 100-149 × 10³/µL; 2 = <100 × 10³/µL; 3 = <50 × 10³/µL.

Laboratory Diagnostic Modalities

All enrolled neonates underwent laboratory evaluation for sepsis at admission. The sepsis screen comprised total leukocyte count, absolute neutrophil count, immature/total neutrophil ratio, micro-ESR, and CRP [28,29].

A sepsis screen was considered positive when at least two of the following abnormal laboratory parameters were present: total leukocyte count < 5000 cells/mm³, absolute neutrophil count below the reference range according to Munro’s chart for term neonates or Mouzinho’s chart for very low birth weight (VLBW) neonates, immature-to-total neutrophil (I/T) ratio > 0.2, micro-ESR > 15 mm in the first hour, or C-reactive protein (CRP) level > 1 mg/dL. The presence of two abnormal parameters in a screen is associated with a sensitivity of 93-100%, specificity of 83%, and positive and negative predictive values of 27% and 100%, respectively, in detecting sepsis.

Blood cultures are considered the gold standard and were obtained via fresh venipuncture (0.5 mL) and used to guide antimicrobial therapy [30].

Lumbar puncture was indicated in early-onset sepsis with positive blood culture or consistent clinical features, and in all late-onset sepsis cases prior to antibiotics.

CSF findings were considered suggestive of meningitis when one or more of the following abnormalities were present: white blood cell (WBC) count > 32 cells/mm³ with predominance of polymorphonuclear cells (PMNs >60%), CSF glucose level < 50-75% of the corresponding serum glucose concentration, CSF protein concentration > 150 mg/dL, or presence of organisms on Gram stain examination [25].

Echocardiography and Hemodynamic Assessment

All neonates diagnosed with sepsis underwent bedside functional two-dimensional (2D) echocardiography within 48 hours of the first positive sepsis screen. The timing of echocardiographic assessment was standardized across participants. Hemodynamically stable neonates were evaluated in the Cardiology outpatient department. Repeat echocardiography was performed in clinically unstable neonates at the discretion of the treating team.

Echocardiographic assessments were performed by trained personnel with experience in neonatal functional echocardiography, using a standardized protocol. Measurements focused on the physiological assessment of preload, myocardial contractility, and afterload. 

Preload was assessed using the inferior vena cava (IVC) collapsibility/distensibility index and superior vena cava (SVC) flow. An increased IVC collapsibility index was suggestive of hypovolemia. SVC flow ≥40 mL/kg/minute was considered indicative of adequate systemic and cerebral perfusion, based on published neonatal reference values.

Cardiac contractility was evaluated using cardiac index (CI) and left ventricular ejection fraction (LVEF). A CI of 2.5-4.0 L/minute/m² was considered normal, while a CI <2.0 L/minute/m² indicated myocardial dysfunction. An LVEF of 54-75% reflected normal global systolic function.

Afterload was assessed using surrogate markers, including systemic vascular resistance and perfusion index (PI). PI values <1.24 in term neonates and <0.88 in preterm neonates were considered indicative of poor peripheral perfusion, as described in previous neonatal studies [30,31].

Statistical analysis

Data were entered into Microsoft Excel spreadsheets (Microsoft Corporation, Redmond, Washington, United States) and analyzed using IBM SPSS Statistics for Windows, version 26 (IBM Corp., Armonk, New York, United States). Continuous variables were expressed as mean ± standard deviation (SD) or range, and categorical variables as frequency (percentage). Comparisons between groups were performed using the Chi-square test for categorical variables and Student’s t-test for continuous variables. A p-value <0.05 was considered statistically significant.

## Results

A total of 288 neonates with sepsis were enrolled and categorized into two groups: (i) those with septic shock (n = 102) and (ii) those without septic shock (n = 186). The mean age of neonates in the septic shock group was 9.45 ± 4.57 days, compared to 10.44 ± 5.14 days in the non-shock group; this difference was not statistically significant (t = 1.625, p = 0.1054). Gender distribution was comparable between the groups, with 46.1% female and 53.9% male neonates in the shock group, versus 40.9% female and 59.1% male neonates in the non-shock group (χ² = 1.298, p = 0.2545). Significant differences were observed in gestational age and birth weight between the two groups. The mean gestational age was significantly lower in neonates with septic shock (231.54 ± 16.84 days) compared to those without shock (241.51 ± 17.48 days; t = 4.689, p < 0.0001). Similarly, the mean birth weight of neonates in the shock group (1751.54 ± 268.41 g) was significantly lower than in the non-shock group (2064.84 ± 241.45 g; t = 10.12, p < 0.0001) (Table 1).

**Table 1:** Demographic profile according to presence or absence of septic shock (N=288) * Significant at p <0.05

Parameter	Sepsis with Septic Shock (n=102)	Sepsis Without Septic Shock (n=186)	Test Statistic	p-value
Age (days), mean ± SD	9.45 ± 4.57	10.44 ± 5.14	t = 1.625	0.105
Female, n (%)	47 (46.1)	76 (40.9)	χ² = 1.298	0.255
Male, n (%)	55 (53.9)	110 (59.1)		
Gestational age (days), mean ± SD	231.5 ± 16.8	241.5 ± 17.5	t = 4.689	<0.001*
Birth weight (g), mean ± SD	1751.5 ± 268.4	2064.8 ± 241.5	t = 10.12	<0.001*

Neonates with septic shock (n = 102) demonstrated significantly higher rates of feeding intolerance (85.3% vs. 22.6%, p = 0.004) and prolonged capillary refill time >3 seconds (85.3% vs. 5.9%, p < 0.001) compared to those without shock (n = 186). Other clinical features, including temperature instability, apnea, abnormal reflexes, and intubation, were more frequent in the shock group but did not reach statistical significance (Table 2).

**Table 2:** Clinical, laboratory, and culture profile of neonates with and without septic shock Data are expressed as mean ± standard deviation (SD) or number (percentage). TLC : total leukocyte count; ANC : absolute neutrophil count ;  ESR : erythrocyte sedimentation rate ; CRP :  C-reactive protein ; CSF : cerebrospinal fluid. * Significant at p <0.05.

Parameter	Sepsis with Septic Shock (n=102)	Sepsis Without Septic Shock (n=186)	Test Statistic	p-value
Clinical Findings				
Feeding Intolerance, n (%)	87 (85.3)	42 (22.6)	χ² = 8.124	0.0044*
Temperature Instability, n (%)	92 (90.2)	63 (33.9)	χ² = 2.745	0.0975
Apnea, n (%)	65 (63.7)	77 (41.4)	χ² = 0.508	0.4760
Abnormal Reflexes, n (%)	54 (52.9)	72 (38.7)	χ² = 1.292	0.2556
Capillary Refill Time >3 sec, n (%)	87 (85.3)	11 (5.9)	χ² = 34.68	<0.0001*
Intubated, n (%)	69 (67.7)	57 (30.7)	χ² = 0.573	0.4492
Laboratory Parameters				
TLC (cells/mm³), mean ± SD	4958.6 ± 1996.7	10842.1 ± 4641.6	t = 12.19	<0.0001*
ANC (cells/mm³), mean ± SD	1953.9 ± 747.7	6381.9 ± 2797.5	t = 15.67	<0.0001*
Immature/Total (IT) Ratio, mean ± SD	0.26 ± 0.03	0.11 ± 0.06	t = 23.67	<0.0001*
Micro-ESR (mm 1st hour), mean ± SD	19.53 ± 6.22	9.09 ± 3.41	t = 18.41	<0.0001*
CRP (mg/dL), mean ± SD	5.47 ± 2.57	0.60 ± 0.20	t = 25.74	<0.0001*
Culture Results				
Biochemical meningitis positive, n (%)	82 (80.4)	33 (17.7)		0.0012*
Blood culture ositive, n (%)	65 (63.7)	24 (12.9)		0.0020*
CSF Culture Positive, n (%)	25 (24.5)	9 (4.8)		0.0798

Laboratory analysis revealed that neonates with shock had markedly lower total leukocyte count (4958.6 ± 1996.7 vs. 10842.1 ± 4641.6 cells/mm³) and absolute neutrophil count (1953.9 ± 747.7 vs. 6381.9 ± 2797.5 cells/mm³) than those without shock (p < 0.0001 for both). In contrast, immature-to-total neutrophil ratio (0.26 ± 0.03 vs. 0.11 ± 0.06), micro-ESR (19.53 ± 6.22 vs. 9.09 ± 3.41 mm in 1st hour), and CRP levels (5.47 ± 2.57 vs. 0.60 ± 0.20 mg/dL) were significantly higher in the shock group (p < 0.0001) (Table 2).

Biochemical evidence of meningitis was detected in 80.39% of neonates with shock compared to 17.74% without shock (p = 0.0012). Blood cultures were positive in 65 neonates with shock and 24 neonates without shock, while CSF cultures were positive in 25 neonates with shock and 9 neonates without shock. These proportions were higher in the shock group, although the difference for CSF culture positivity did not reach statistical significance (p = 0.0798) (Table 2).

 Echocardiographic assessment revealed significant cardiovascular compromise in neonates with septic shock (n = 102) compared to those without shock (n = 186). Neonates with shock exhibited markedly lower mean SVC flow (33.8 ± 16.4 vs. 82.6 ± 17.5 ml/kg/min), cardiac index (1.84 ± 0.76 vs. 2.72 ± 0.78 L/minute/m²), LVEF (45.6 ± 8.7 vs. 64.9 ± 8.7%), diastolic arterial blood pressure (31.7 ± 5.1 vs. 42.7 ± 5.2 mmHg), and perfusion index (0.65 ± 0.08 vs. 1.18 ± 0.44) (all p < 0.0001). Conversely, mean IVC collapsibility (40.4 ± 6.0 vs. 22.1 ± 6.3%) and IVC distensibility (22.7 ± 4.3 vs. 16.1 ± 5.7%) were significantly higher in the shock group. A greater proportion of neonates with shock fell below critical thresholds for these parameters, highlighting compromised cardiac function and systemic perfusion in the presence of shock (Table 3).

**Table 3:** Echocardiographic parameters in neonates with and without septic shock SVC: superior vena cava; IVC: inferior vena cava; POG: period of gestation; LVEF: left ventricular ejection fraction * significant at p <0.05

Parameter	Shock (n=102)	Without Shock (n=186)	Test Statistic	p-value
SVC Flow (ml/kg/min), mean±SD	33.84 ± 16.36	82.59 ± 17.52	t = 23.11	<0.0001*
≤40 ml/kg/min, n (%)	76 (74.5)	10 (5.9)	χ² = 150.3	<0.0001*
IVC Collapsibility (%) (non-intubated, n=162)	40.38 ± 6.01	22.08 ± 6.28	t = 24.01	<0.0001*
>36%, n (%)	20 (60.6)	10 (7.8)	χ² = 14.30	<0.0001*
IVC Distensibility (%) (intubated, n=126)	22.68 ± 4.25	16.09 ± 5.68	t = 10.25	<0.0001*
>18%, n (%)	47 (68.1)	8 (14.0)	χ² = 74.42	<0.0001*
Cardiac Index (L/min/m²), mean±SD	1.84 ± 0.76	2.72 ± 0.78	t = 9.24	<0.0001*
<2.5 L/min/m², n (%)	90 (88.2)	18 (9.7)	χ² = 173.5	<0.0001*
LVEF (%), mean±SD	45.60 ± 8.69	64.87 ± 8.69	t = 18.00	<0.0001*
<55%, n (%)	73 (71.6)	14 (7.5)	χ² = 128.1	<0.0001*
Diastolic Arterial BP (mmHg), mean±SD	31.65 ± 5.08	42.74 ± 5.17	t = 17.52	<0.0001*
<5th centile (POG), n (%)	87 (85.3)	20 (10.8)	χ² = 156.8	<0.0001*
Perfusion Index, mean±SD	0.65 ± 0.08	1.18 ± 0.44	t = 12.05	<0.0001*
<1.0, n (%)	95 (93.1)	32 (17.2)	χ² = 130.7	<0.0001*

Among neonates with septic shock (n = 102), the majority (80.4%) required inotropes or vasopressors, while 19.6% improved with fluid boluses alone. Of those receiving pharmacological support (n = 82), most (57.3%) required two agents, 24.4% needed only one, and 18.3% required three or more, reflecting varying degrees of hemodynamic instability. Regarding the types of agents used, dobutamine combined with norepinephrine was the most common regimen (37.8%), followed by dobutamine alone (15.9%), dobutamine + epinephrine (19.5%), and the triple combination of dobutamine + norepinephrine + epinephrine (18.3%). Standalone norepinephrine and epinephrine were less frequently employed (Table 4).

**Table 4:** Use of inotropes/vasopresssors in neonate with septic shock (N =102)

Parameter	Frequency (Percentage)
Use of Inotropes/Vasopressors	
Yes	82 (80.39)
No	20 (19.61)
Number of Inotropes/Vasopressors Used (n=82)	
1	20 (24.39)
2	47 (57.32)
≥3	15 (18.29)
Type of Inotropes/Vasopressors Used (n=82)	
Dobutamine	13 (15.85)
Norepinephrine	5 (6.10)
Epinephrine	2 (2.44)
Dobutamine + Norepinephrine	31 (37.80)
Dobutamine + Epinephrine	16 (19.51)
Dobutamine + Norepinephrine + Epinephrine	15 (18.29)

Neonates with septic shock demonstrated significantly worse outcomes compared to those without shock. Among the shock group, 46.1% of neonates died, while 48.0% were successfully discharged, and 5.9% left against medical advice (LAMA). In contrast, the majority of neonates without shock were discharged (86.0%), with a mortality rate of 9.1% and 4.8% LAMA. These findings underscore the markedly higher risk of mortality and adverse outcomes associated with neonatal septic shock (p < 0.0001).

Blood and cerebrospinal fluid (CSF) cultures of neonates with septic shock revealed a predominance of gram-negative organisms. In blood cultures (n = 89), *Klebsiella pneumoniae* was the most frequently isolated pathogen (42.7%), followed by *Acinetobacter baumanii* (32.6%). Gram-positive organisms, including *Staphylococcus haemolyticus* (16.9%), *Staphylococcus hominis* (4.5%), and *Staphylococcus epidermidis* (3.4%), were identified less commonly. CSF cultures (n = 34) were dominated by *A. baumanii*, accounting for 79.4% of isolates, while other pathogens collectively represented 20.6% of cases (Table 5). These findings highlight the predominance of gram-negative infections in neonates with septic shock and underscore the importance of targeted antimicrobial therapy.

**Table 5:** Microbiological profile of neonates with septic shock CSF: cerebrospinal fluid

Culture Type	Pathogen	Frequency (Percentage)
Blood Culture (n=89)	Klebsiella pneumoniae	38 (42.70)
	Acinetobacter baumanii	29 (32.58)
	Staphylococcus haemolyticus	15 (16.85)
	Staphylococcus hominis	4 (4.49)
	Staphylococcus epidermidis	3 (3.37)
CSF Culture (n=34)	Acinetobacter baumanii	27 (79.41)
	Other pathogens	7 (20.59)

## Discussion

This prospective observational study was conducted to evaluate the prevalence of septic shock among neonates admitted with sepsis and to assess their hemodynamic status using functional echocardiography.

In this study, the prevalence of septic shock among sepsis-positive neonates was 35.41%. The mean age of neonates with shock was 9.45 ± 4.57 days, which was not significantly different from that of neonates without shock (10.44 ± 5.14 days). However, neonates with shock had significantly lower gestational age (231.54 ± 16.84 days vs. 241.51 ± 17.48 days; p < 0.0001) and birth weight (1751.54 ± 268.41 g vs. 2064.84 ± 241.45 g; p < 0.0001) than those without shock, indicating that prematurity and low birth weight are independent risk factors for septic shock. Johnston and de Waal demonstrated that preterm infants with early-onset sepsis, particularly those of lower gestational age and birth weight, had a markedly higher risk of progression to septic shock and mortality, highlighting the vulnerability of immature cardiovascular physiology in this population [32]. Similarly, Pawale et al. reported that preterm neonates with shock exhibit complex and overlapping hemodynamic derangements detectable on echocardiography, emphasizing that immature myocardial function and circulatory adaptation contribute significantly to shock development in this group [33]. Extending these observations to term neonates, Bandyopadhyay et al. showed that functional echocardiography parameters such as LVEF, fractional shortening, myocardial performance index, and superior vena cava flow correlate significantly with clinical indicators of shock severity, reinforcing the utility of echocardiography as an adjunct to clinical assessment in neonatal shock [34]. Collectively, these studies support the present findings that gestational maturity and birth weight strongly influence susceptibility to septic shock and underscore the importance of early echocardiographic evaluation for timely identification and targeted management of hemodynamic instability in both preterm and term neonates [32-34].

Clinically, neonates with shock showed significantly higher rates of feeding intolerance (85.29%) and prolonged capillary refill time (>2 sec, 85.29%) compared to those without shock (22.58% and 5.91%, respectively), confirming their utility as early clinical markers for impending shock. Other findings, such as apnea, abnormal reflexes, temperature instability, and intubation, were more frequent in neonates with shock, though not all reached statistical significance. These findings are concordant with earlier studies describing feeding intolerance, delayed capillary refill, lethargy, abnormal reflexes, and respiratory distress as common clinical manifestations of neonatal sepsis and shock [35-37]. Notably, the incidence of these clinical signs in our cohort was higher than previously reported, which may be attributable to the referral bias inherent to our tertiary care center that predominantly manages preterm and low-birth-weight neonates born to critically ill mothers. Collectively, these observations emphasize that simple clinical markers-particularly feeding intolerance and prolonged capillary refill time-remain valuable tools for early recognition of shock in septic neonates, especially in resource-limited settings where advanced hemodynamic monitoring may not be immediately available [35-37].

Laboratory parameters indicated that neonates with shock had significantly lower mean TLC (4958.60 ± 1996.71 cells/mm³) and ANC (1953.85 ± 747.65 cells/mm³) compared to neonates without shock (TLC: 10842.08 ± 4641.62 cells/mm³; ANC: 6381.93 ± 2797.48 cells/mm³). Conversely, IT-ratio (0.26 ± 0.03), micro ESR (19.53 ± 6.22 mm), and CRP (5.47 ± 2.57 mg/dL) were higher in the shock group (p < 0.0001 for all), consistent with prior studies emphasizing the role of immature-to-total neutrophil (I/T) ratio as an early marker of early-onset sepsis [38-40]. Culture positivity was also higher among neonates with shock: biochemical meningitis (80.39%), blood culture (63.72%), and CSF culture (24.50%), with *K. pneumoniae* (42.70%) and *A. baumanii* (32.58%) predominating in blood cultures, and *A. baumannii* accounting for 79.41% of CSF isolates. These results indicate a diverse microbial etiology, with higher positivity rates than other reports, likely due to repeated sepsis screening in deteriorating patients [41,42].

Echocardiographic assessment in the present study revealed significant hemodynamic compromise in neonates with septic shock. Neonates with shock had markedly lower mean SVC flow (33.84 ± 16.36 mL/kg/minute), cardiac index (1.84 ± 0.76 L/minute/m²), LVEF (45.60 ± 8.69%), diastolic arterial pressure (31.65 ± 5.08 mmHg), and perfusion index (0.65 ± 0.08), along with significantly higher IVC collapsibility (40.38 ± 6.01%) and IVC distensibility index (22.68 ± 4.25%) compared with neonates without shock (p < 0.0001 for all). These findings indicate reduced preload, impaired cardiac output, and compromised systemic perfusion, reflecting the coexistence of systolic and diastolic myocardial dysfunction in neonatal septic shock. Similar observations have been reported in pediatric sepsis, where Sanfilippo et al. demonstrated that although conventional systolic indices such as LVEF may not consistently predict mortality, echocardiographic parameters reflecting diastolic dysfunction are significantly associated with adverse outcomes, highlighting the importance of early myocardial relaxation abnormalities in septic states [43]. Additionally, altered ventricular filling dynamics and right ventricular loading conditions, as described in critical care studies on acute right ventricular dysfunction, may further aggravate low cardiac output and systemic hypoperfusion in septic shock [44]. Supporting these findings, Sankar et al. reported a high prevalence (41.1%) of diastolic dysfunction in children with fluid-refractory septic shock, with higher mortality (43%) among affected patients, emphasizing the prognostic and therapeutic value of early bedside echocardiographic assessment in guiding hemodynamic management [45].

Pharmacologically, a substantial proportion of neonates with shock in the present study required vasoactive support, with 80.39% receiving inotropes and/or vasopressors. Among these, the majority required escalation to two agents (57.32%), and the most frequently used combination was dobutamine with norepinephrine (37.80%), reflecting the need for both inotropic support and vasopressor augmentation in severe septic shock. This pattern is consistent with existing literature emphasizing early and aggressive pharmacological management to counteract sepsis-induced myocardial depression, vasodilatation, and impaired tissue perfusion [46,47]. Despite such interventions, outcomes among neonates with shock remained poor, with significantly higher mortality (46.07% vs. 9.13%) and lower discharge rates (48.03% vs. 86.02%) compared with neonates without shock, underscoring the severity and lethality of neonatal septic shock. Similar high mortality rates have been reported in preterm infants with early-onset sepsis requiring inotropic support, particularly among those progressing to septic shock and exhibiting echocardiographic evidence of myocardial dysfunction [33]. Likewise, studies from resource-limited settings have demonstrated persistently elevated mortality in neonates with severe sepsis and shock, especially in association with prematurity, low birth weight, and hemodynamic instability [37]. Collectively, these findings highlight that, despite prompt and escalated vasoactive therapy, neonatal septic shock continues to carry a high risk of adverse outcomes, reinforcing the need for early identification, targeted hemodynamic assessment, and individualized management strategies.

Overall, this study highlights the multifactorial nature of neonatal septic shock, encompassing prematurity, clinical manifestations, inflammatory response, microbial etiology, and complex hemodynamic alterations. Early identification using clinical and laboratory indicators, combined with functional echocardiography, may facilitate timely and individualized hemodynamic management.

Limitations and future recommendations

The study has certain limitations. Being a single-center study, the findings may not be generalizable to all settings. Invasive monitoring of systemic vascular resistance was not feasible due to resource constraints. Functional echocardiography was performed following the first positive sepsis screen rather than serially, which may have introduced variability in hemodynamic assessment. Additionally, the absence of multivariable adjustment limits the assessment of independent associations. Potential observer and measurement variability and the possibility of confounding factors influencing outcomes are also limiting factors. Despite these limitations, the prospective design and comprehensive hemodynamic evaluation provide valuable insights into neonatal septic shock.

Future multicenter studies with standardized protocols, serial echocardiographic assessment, and long-term follow-up are warranted to improve generalizability and better define optimal hemodynamic management strategies in neonatal sepsis and septic shock.

## Conclusions

Neonatal sepsis is frequently complicated by septic shock, which is associated with significant hemodynamic instability and high mortality. In this study, prematurity and low birth weight were identified as independent risk factors for septic shock in neonates with sepsis. Clinical indicators such as feeding intolerance and prolonged capillary refill time, along with laboratory markers including leukopenia, elevated I/T ratio, CRP, and micro-ESR, were strongly associated with shock and may aid in early recognition of circulatory compromise.

Functional echocardiography revealed significant impairments in preload, myocardial contractility, and systemic perfusion in neonates with shock, supporting its role as a valuable bedside tool for early hemodynamic assessment. Most affected neonates required inotropic or vasopressor support, often in combination, and mortality remained substantially higher in the shock group despite aggressive management. Early integration of clinical, laboratory, and echocardiographic assessment may enable targeted hemodynamic interventions and improve outcomes, while multicenter studies are needed to further validate these findings.
